# DEM-Based Investigation of Sand Mixing Ratio and Recoating Speed Effects on Recoating Performance and Mechanical Properties in 3D Sand Printing

**DOI:** 10.3390/ma19030473

**Published:** 2026-01-24

**Authors:** Guili Gao, Jialin Guo, Jie Liu, Dequan Shi, Huajun Zhang

**Affiliations:** 1 School of Mechanical and Energy Engineering, Shanghai Technical Institute of Electronics and Information, Shanghai 201411, China; gao-gui@163.com; 2 Prismlab China Ltd., Shanghai 201615, China; jason@prismlab.com; 3 School of Materials and Chemistry, University of Shanghai for Science and Technology, Shanghai 200093, China; 243403189@st.usst.edu.cn; 4 Shanghai Changxing Ocean Laboratory, School of Materials Science and Engineering, Shanghai Jiao Tong University, Shanghai 200240, China

**Keywords:** 3D sand printing, discrete element method, ratio of silica sand to ceramsite sand, recoating speed, mechanical properties

## Abstract

Based on the discrete element method (DEM), a sand particle contact force model and a motion model for the 3D sand printing (3DSP) process were developed. By accounting for the viscous support force and contact force between sand particles, and gravity acting on each individual sand particle, the displacement of sand particles was calculated, enabling the simulation of the 3DSP process using sand particle ensembles. Furthermore, the effects of the ratio of silica sand to ceramsite sand and the recoating speed on sand-recoating performances and mechanical properties were investigated. Irregularly shaped sand particles (primarily silica sand) were constructed via the multi-sphere filling method. The simulation was performed on a virtual sand-recoating device (180 mm in length, 100 mm in width, 70 mm in height) with reference to the EXONE S-MAX printer. Meanwhile, the EXONE S-MAX was utilized to print the bending samples for experimental validation. Simulation and experimental results indicate that as the ratio increases, the porosity first decreases and then increases, whereas mechanical properties exhibit an initial increase followed by a decrease. At a ratio of 3:7, the porosity reaches a minimum of 21.3%; correspondingly, the shear force of bonding bridges peaks at 908 mN, and the bending strength of specimens attains a maximum of 2.87 MPa. With the increasing recoating speed, the porosity rises consistently, while the shear force of bonding bridges and the bending strength of specimens first increase and then decrease, which is primarily attributed to the penetration behavior of the binder under capillary force. At a recoating speed of 160 mm·s^−1^, the shear force of bonding bridges reaches its maximum, and the specimens achieve a maximum bending strength of 2.89 MPa. The simulation results are well-validated by the experiments. The DEM-based simulation method proposed in this study offers a practical and convenient tool for parameter optimization in 3DSP process.

## 1. Introduction

Rapid prototyping technology, developed in the mid-to-late 1980s, is an additive manufacturing technique rooted in the “discretization/accumulation” principle. It offers distinct advantages such as shortened manufacturing cycles, reduced costs, and is free from constraints imposed by the model complexity [1,2], and is also referred to as 3D printing, additive manufacturing, rapid manufacturing, growth manufacturing, or layered manufacturing [3,4,5]. Rapid prototyping technology enables the fabrication of three-dimensional solid models with diverse geometries through a unified, automated workflow. Notably, the difficulty of its forming process is largely independent of the shape or structural complexity of the target solid [6], which overcomes the limitations of traditional modeling and ensures flexibility in part design and iterative optimization [7]. Since the 1990s, 3D printing technology has been applied in the traditional casting field [8]. In contrast to conventional mold-dependent production, 3D sand printing (3DSP) features a simpler process that eliminates the need for mold and core box fabrication, thereby saving manufacturing time and reducing costs [9,10,11].

The 3DSP technology directly fabricates physical objects from computer-based geometric descriptions of part designs. Specifically, it is a rapid prototyping technique that employs an inkjet head to selectively eject binders onto a powder bed, followed by layer-by-layer printing and accumulation to finally obtain the mold [12].

In casting processes, the precision and quality of parts are closely related to the performance of sand molds, which is directly influenced by 3DSP process parameters. Thus, research on the optimization of process parameters plays a critical role in enhancing sand mold precision and performance. Some researchers have investigated the relationship between the characteristics of 3DSP samples and process parameters [13,14,15]. Vaezi et al. [16] investigated the effects of layer thickness and binder saturation level on the mechanical properties, surface quality, and dimensional accuracy of 3D-printed samples. Their findings revealed that binder saturation level and layer thickness exert significant impacts on the tensile strength, flexural strength, and surface uniformity of the samples. The increased binder saturation level improved tensile and flexural strength but reduced dimensional accuracy and surface uniformity, while thicker layers decreased tensile strength yet enhanced flexural strength and surface uniformity. Aslan et al. [17] developed a novel binder jet 3D printer, and examined the effects of three parameters—printhead feed rate, curing agent ratio, and sand particle size—on the flexural strength, dimensional tolerance, and porosity of samples. Their results showed that the sample porosity ranged from 38.69% to 56.65%, the maximum flexural strength reached 0.38 MPa, and the optimal dimensional tolerance for the diameter of cylindrical samples was 0.6 mm. Gao et al. [18] selected activator content, resolution X, layer thickness, and recoating speed as influencing factors, with flexural strength and dimensional deviation in samples as evaluation indicators. Through orthogonal experiments, they identified resolution X and layer thickness as the primary factors governing sand mold performance. Minimizing both parameters can yield the highest flexural strength, and the optimal process parameters were determined as 0.19% activator content, 0.1 mm resolution X, 0.28 mm layer thickness, and 210 mm·s^−1^ recoating speed. Sivarupan and Coniglio [19,20] studied the effects of 3D printing process parameters on sand mold bending strength and air permeability. They found that at low recoating speeds, higher printing resolution reduces air permeability of sand mold without affecting its strength. At high recoating speeds, however, higher resolution improves the strength by effectively connecting sand particles and their gaps, and a maximum gap area of 46% was found to enhance sand mold air permeability.

The 3DSP technology in foundry integrates the forming advantages of 3D printing with the performance benefits of traditional casting. Nevertheless, casting quality is influenced by numerous process parameters, and many experiments are required in production to obtain the optimal process parameters [21], leading to high costs. Therefore, prior to fabricating molds via 3D printing, modeling and simulation can facilitate optimized design with lower costs and shorter development cycles [22,23]. Currently, the discrete element method (DEM) is widely used to study inter-particle interactions. Xu et al. [24] employed DEM to model the spreading behavior of cohesive sand powder, focusing on its spreadability and mechanical jamming. Their results indicated that particle size segregation occurred before particles entered the gap between the scraper and substrate. Although the size of large particles is smaller than the gap height, they are more difficult to spread to the substrate surface due to the jamming effect. Markl et al. [25] proposed an effective coupling method between the classical discrete element approach and a grid-based solver for simulating powder layer fusion. After verifying the method’s validity, they applied it to simulate the selective electron beam melting process. Haeri et al. [26] used DEM to simulate the spreading of rod-shaped particles and analyzed the effects of particle shape and operating conditions on powder bed quality. They observed that larger particle aspect ratios or higher spreader translational speeds degraded powder bed quality, manifesting as reduced surface quality, increased surface roughness, and lower solid volume fraction. Lee et al. [27] developed a DEM-based model that can calculate mechanical contact forces and moments between individual particles, and simulated the accumulation dynamics of two powders with different particle sizes in additive manufacturing. Experiments confirmed that this powder accumulation model is applicable to practical binder jet additive manufacturing scenarios.

Based on the previous research, it is evident that 3DSP parameters exert a significant influence on casting quality. However, existing studies still exhibit notable gaps. First, although the DEM has been applied to investigate powder spreading and particle interactions in 3DSP, most studies focus on single-type sand particles (e.g., spherical silica sand) or an individual parameter (e.g., particle size or layer thickness), with insufficient attention paid to the effects of mixed sand (e.g., irregular silica sand and spherical ceramsite sand) on recoating performance and mechanical properties. Second, previous experimental or simulation studies rarely combine macroscopic recoating behavior with microscopic bonding bridge characteristics, resulting in an incomplete understanding of the inherent relationship between process parameters and sand mold strength. To address these gaps, this study employs DEM to establish a mathematical model for the force state of sand particles during the 3DSP recoating process. By considering the supporting force, contact force, and gravity acting on each particle, the displacement of sand particles is calculated. Combined with the sand particle model, recoater model, and workbench model constructed using SolidWorks 2024 and EDEM 2024, the simulation of the 3DSP recoating process based on sand particle ensemble is realized. Additionally, the effects of the mixing ratio of silica sand to ceramsite sand and the recoating speed are simulated and analyzed to determine the optimal parameter. To verify the reliability and accuracy of the established simulation model, the simulation results are compared and discussed with the experimental measurements.

Through this study, it is clarified that the sand mixing ratio determines the initial particle packing, while the recoating speed regulates the binder penetration depth via capillary function. They jointly determine the formation process of bonding bridges, which is of great significance for a comprehensive understanding of how to optimize particle packing and binder distribution simultaneously.

## 2. Establishment of Sand-Recoating Model Based on DEM

### 2.1. Contact Mechanics Model of Sand Particles

In the recoating-sand process of 3DSP, the motion of sand particles inevitably induces mutual collisions, which in turn give rise to forces and displacements. The entire process can be characterized by the kinematics and relative positional relationships of all sand particles. For this study, mixed sand—formulated by silica sand and ceramsite sand at a predefined ratio—was utilized. Ceramsite sand features an angularity coefficient approaching 1, rendering it approximately spherical, while silica sand possesses a considerably larger angularity coefficient and is therefore classified as non-spherical. Consequently, the 3DSP simulation process involves a mixture of spherical and non-spherical particles, thus necessitating the establishment of a contact mechanics model for sand particles in advance.

Depending on the distinct contact behaviors between particles, the DEM primarily employs two fundamental approaches: the hard-sphere method and the soft-sphere method [28]. The hard-sphere method idealizes particle contact as an instantaneous collision, in which inter-particle contact forces are neglected, and only the post-collision velocities of particles are taken into consideration. By contrast, the soft-sphere method incorporates collisions involving multiple particles, with a sustained duration of interaction between the particles.

**Figure 1:**
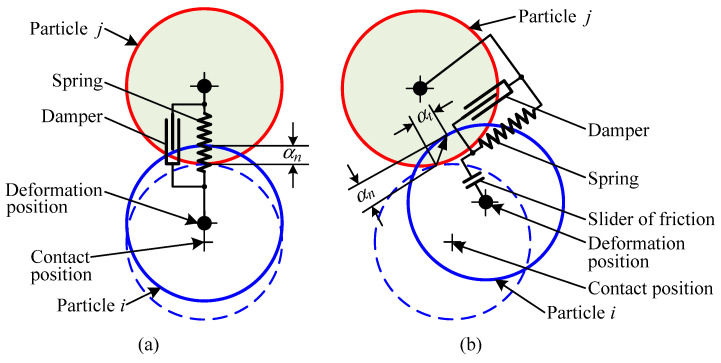
Contact mechanics model for sand particles during 3DSP sand-recoating developed based on the soft-sphere method: (**a**) normal mechanical model, (**b**) tangential mechanical model. The blue dashed line indicates the original positions of the particle *i*.

Given the inherent characteristic of extensive inter-particle interactions in the 3DSP process, the soft-sphere method was selected as the foundational framework for modeling sand particle contact in this study. The developed contact mechanics model for sand particles is illustrated in Figure 1. Within this model, the normal force between sand particles is simplified as a combination of a spring and a damper, whereas the tangential force is represented as an assembly comprising a spring, a damper, and a slider. In Figure 1, the dashed line denotes the relative position of two sand particles (Particle *i* and Particle *j*) at the initial contact instant. Driven by external forces or inertial forces, Particle *i* initiates contact with Particle *j*. As the two particles undergo relative motion, the surfaces of the contacting particles exhibit slight deformation. However, the magnitude of this deformation is extremely small and thus negligible. Accordingly, only the translational and rotational motions of the two particles are considered, which in turn give rise to tangential displacement ($\alpha_{t}$) and normal overlap ($\alpha_{n}$) between the sand particles. Following the introduction of parameters such as damping coefficient (*c*) and elastic coefficient (*k*), the contact force between sand particles can be computed from the particles’ tangential displacement and normal overlap, in accordance with the fundamental motion equations governing sand particles.

### 2.2. Basic Motion Equations of Sand Particles

**Figure 2:**
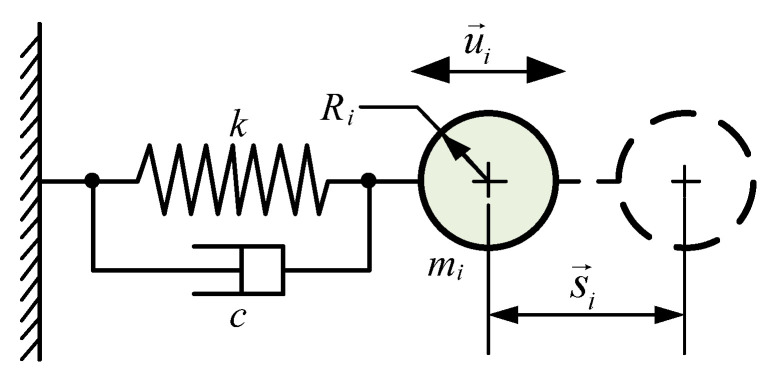
Equivalent spring oscillator system model for sand particle motion.

Based on the developed contact mechanics model for sand particles, the contact behaviors—both between sand particles and mechanical structures—can be characterized using vibrational motion equations. The inter-particle contact process of sand can be equivalent to a damped vibration system of a spring oscillator, as illustrated in Figure 2. Its fundamental motion equation is expressed as follows:

(1) $$\left\{\begin{array}{l} m_{i}\frac{d^{2}{\vec{s}}_{i}}{dt^{2}}=-c{\vec{u}}_{i}+k{\vec{s}}_{i}\\ \vec{u}=\frac{d{\vec{s}}_{i}}{dt} \end{array}\right.$$

where $m_{i}$ denotes the mass of sand particle *i* within the damped vibration system of the spring oscillator; ${\vec{s}}_{i}$ represents the displacement of sand particle *i* relative to its equilibrium position, with ${\vec{s}}_{i}={\vec{s}}_{i}\left(t\right)$ which encompasses the tangential displacement $\alpha_{t}$ and normal overlap $\alpha_{n}$; ${\vec{u}}_{i}$ is the velocity vector of sand particle *i* at time *t*, with ${\vec{u}}_{i}={\vec{u}}_{i}(t)$; *c* and *k* are the damping coefficient and elastic coefficient of the spring oscillator (i.e., sand particle *i*), respectively.

As inferred from Equation (1), the viscous drag force exerted on a sand particle is proportional to the magnitude of its velocity, whereas the restoring force is proportional to the magnitude of its displacement but acts in the opposite direction. As a result, the total energy of the sand particle dissipates gradually until the particle reaches a stable state.

Using the recurrence formula of the second-order Taylor series, the motion equations describing the displacement and velocity of sand particle *i* are expressed as follows:

(2) $$\left\{\begin{array}{l} s_{i}\left(t+\Delta t\right)\approx 2s_{i}\left(t\right)-s_{i}\left(t-\Delta t\right)+\frac{1}{m_{i}}F_{i}\left(t\right)\Delta t^{2}\\ u_{i}\left(t\right)\approx \frac{1}{2\Delta t}[s_{i}\left(t+\Delta t\right)-s_{i}\left(t-\Delta t\right)] \end{array}\right.$$

where $s_{i}(t)$, $s_{i}(t+\Delta t)$ and $s_{i}(t-\Delta t)$ denote the displacements of sand particle *i* at times *t*, *t* + Δ*t* and *t* − Δ*t*, respectively; $F_{i}\left(t\right)$ represents the total force acting on sand particle *i* at time *t*, which depends on the physical properties and instantaneous motion state of sand particle *i*; Δ*t* is the time step, which can be defined as follows [29]:

(3) $$\Delta t=\pi \frac{R_{i}}{0.165\nu_{i}+0.876}{\left(\frac{\rho_{i}}{G_{i}}\right)}^{\frac{1}{2}}$$

where $G_{i}$ is the shear modulus of sand particle *i*; $\rho_{i}$ is the density of sand particle *i*; $\nu_{i}$ denotes the Poisson’s ratio of sand particle *i*; $R_{i}$ is the radius of sand particle *i*.

As deduced from Equation (2), a detailed analysis of the total force $F_{i}\left(t\right)$ on the sand particle is essential for performing iterative calculations based on the particle’s displacement and velocity.

### 2.3. Contact Force Equations of Sand Particles

Silica sand belongs to non-spherical particles, and the calculation of its contact force is far more complex than that of spherical ceramsite sand. One approach is to determine whether contact occurs between two adjacent sand particles of a specific shape by solving the particles’ fundamental mathematical equations. However, this method involves considerable complexity in solving the mathematical equations [30]. An alternative approach represents non-spherical sand particles as a collection of sub-spheres termed “spherical elements”. These spherical elements may overlap or remain non-overlapping, but the position of each spherical element relative to other spherical elements within the same sand particle remains fixed. Thus, the contact mechanics problem of non-spherical sand particles can be resolved through basic contact calculations between spherical elements [31,32]. Contact between sand particles is identified if the distance between the centers of two contacting spherical elements is less than or equal to the sum of their radii. In such cases, the total force $F_{i}\left(t\right)$ acting on a non-spherical sand particle equals the vector sum of the resultant forces exerted on its spherical elements. Consequently, the multi-sphere method—where irregularly shaped sand particles are filled with spherical elements—can be employed to represent such particles.

**Figure 3:**
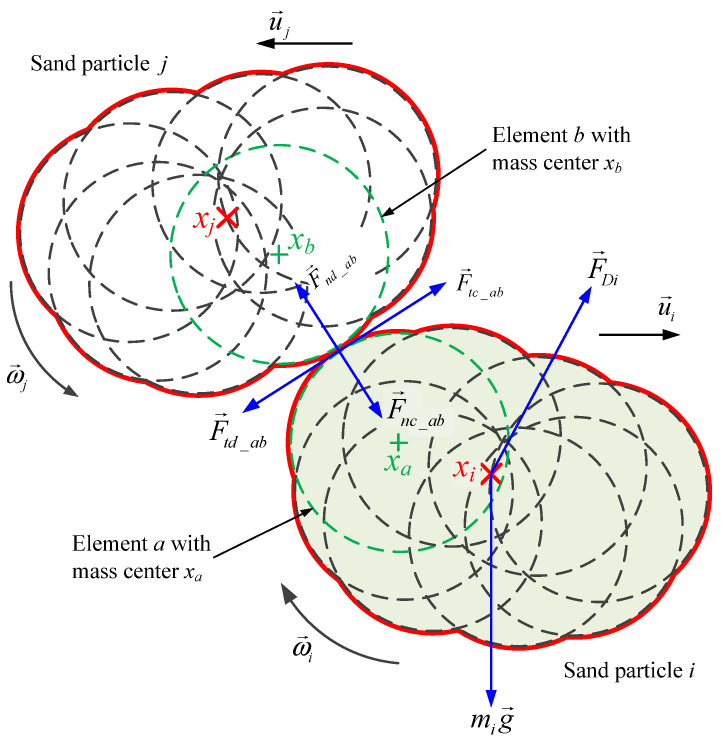
Two-dimensional schematic diagram of forces acting on non-spherical sand particles *i* and *j*.

Figure 3 presents a schematic illustration of the interaction forces when two non-spherical particles (silica sand particles *i* and *j*) are in contact. For the specific modeling of sand particles, 15 spherical elements were utilized in this study. For simplicity, Figure 3 schematically depicts the force distribution using eight spherical elements. The forces include the gravitational force $m_{i}\vec{g}$ of sand particle *i*, the drag force ${\vec{F}}_{Di}$, and the contact force between elements *a* and *b*. The contact force between elements *a* and *b* consists of the tangential contact force ${\vec{F}}_{tc\_ab}$, tangential damping force ${\vec{F}}_{td\_ab}$, normal contact force ${\vec{F}}_{nc\_ab}$, and normal damping force ${\vec{F}}_{nd\_ab}$. $x_{i}$ and $x_{j}$ denote the centers of mass of sand particles *i* and *j*, respectively, while $x_{a}$ and $x_{b}$ represent the centers of mass of elements *a* and *b* which belong to sand particles *i* and *j*, respectively.

Obviously, when the centers of mass of all elements within a sand particle coincide, the sand particle transforms into a spherical particle, corresponding to ceramsite sand. The size of the sand particle is governed by the radii of its constituent elements, whereas the particle’s shape is determined by the positions of the elements’ centers of mass. Thus, spherical sand particles can be regarded as a special case of non-spherical sand particles. For the analysis of spherical sand particles (ceramsite sand), it is only necessary to treat elements *a* and *b* as sand particles *i* and *j*, respectively. Consequently, the subsequent contact force analysis will be performed based on non-spherical sand particles.

The translational velocity and rotational velocity of sand particle *i* adhere to Newton’s laws of motion with respect to the applied forces, which can be expressed as follows:

(4) $$\left\{\begin{array}{l} m_{i}\frac{d{\vec{u}}_{i}}{dt}=m_{i}\vec{g}+{\vec{F}}_{Ci}+{\vec{F}}_{Di}\\ I_{i}\frac{d{\vec{\omega }}_{i}}{dt}=T_{i}=\sum_{j=1}^{N}\left({\vec{T}}_{tij}+{\vec{T}}_{nij}\right) \end{array}\right.$$

where $m_{i}$ is the mass of sand particle *i*; $\vec{u}$ and ${\vec{\omega }}_{i}$ denote the translational velocity and rotational velocity of sand particle *i*, respectively; ${\vec{F}}_{Ci}$ is the total contact force acting on sand particle *i*; ${\vec{F}}_{Di}$ represents the drag force exerted on sand particle *i*; $I_{i}$ is the moment of inertia of sand particle *i*; ${\vec{T}}_{tij}$ and ${\vec{T}}_{nij}$ are the torques induced by the tangential force and normal force between sand particle *i* and sand particle *j*, respectively; *N* denotes the number of sand particles in contact with sand particle *i*; *t* is time.

In accordance with Hertzian contact theory [33], the total contact force exerted on sand particle *i* equals the sum of the contact forces between all element–element pairs within sand particle *i*, and the contact forces between these elements and the platform. This relationship is expressed as follows:

(5) $${\vec{F}}_{Ci}=\sum_{a=1}^{N_{i}}\sum_{b=1}^{N_{j}}\left({\vec{F}}_{tc\_ab}+{\vec{F}}_{nc\_ab}+{\vec{F}}_{td\_ab}+{\vec{F}}_{nd\_ab}\right)$$

where $N_{i}$ denotes the number of contacted spherical elements within sand particle *i*; $N_{j}$ denotes the number of spherical elements in sand particle *j* that are in contact with element *a*; ${\vec{F}}_{tc\_ab}$ and ${\vec{F}}_{nc\_ab}$ represent the tangential contact force and normal contact force between element *a* and element *b*, respectively; and ${\vec{F}}_{td\_ab}$ and ${\vec{F}}_{nd\_ab}$ are the tangential damping force and normal damping force between element *a* and element *b*, respectively.

Based on the Hertz–Mindlin no-slip model which is derived from classical Hertz theory [34,35], the tangential contact force ${\vec{F}}_{tc\_ab}$ between elements *a* and *b* is proportional to the tangential overlap, and can be expressed as follows:

(6) $$\left\{\begin{array}{l} {\vec{F}}_{tc\_ab}=-k_{tab}\alpha_{tab}\vec{n}\\ k_{tab}=8{\left(\frac{1}{R_{a}}+\frac{1}{R_{b}}\right)}^{-\frac{1}{2}}{\left(\frac{1-\nu_{a}^{2}}{G_{a}}+\frac{1-\nu_{b}^{2}}{G_{b}}\right)}^{-1}\alpha_{nab}^{\frac{1}{2}} \end{array}\right.$$

where $\alpha_{tab}$ denotes the tangential overlap between elements *a* and *b*; $\vec{n}$ represents the unit vector from the center of mass of element *a* to that of element *b*; $k_{tab}$ is the tangential elastic coefficient (or stiffness coefficient) of elements *a* and *b*; $R_{a}$ and $R_{b}$ are the radii of elements *a* and *b*; $\nu_{a}$ and $\nu_{b}$ denote the Poisson’s ratios of elements *a* and *b*; $G_{a}$ and $G_{b}$ represent the shear moduli of elements *a* and *b*; $\alpha_{nab}$ is the normal overlap between elements *a* and *b*.

Since elements *a* and *b* are sub-spheres constituting sand particles *i* and *j*, respectively, and elements inherit the same physical properties as their parent sand particles, the Poisson’s ratios and shear moduli of elements *a* and *b* are equal to those of sand particles *i* and *j*. That is $\nu_{a}=\nu_{i}$, $\nu_{b}=\nu_{j}$, $G_{a}=G_{i}$ and $G_{b}=G_{j}$.

The relationship between the normal contact force ${\vec{F}}_{nc\_ab}$ and the normal overlap $\alpha_{nab}$ between elements *a* and *b* is expressed as follows [36]:

(7) $$\left\{\begin{array}{l} {\vec{F}}_{nc\_ab}=-k_{nab}\alpha_{nab}\vec{n}\\ k_{nab}=\frac{4}{3}\left(\frac{1-\nu_{a}^{2}}{E_{a}}\right.+{\left.\frac{1-\nu_{b}^{2}}{E_{b}}\right)}^{-1}{\left(\frac{1}{R_{a}}+\frac{1}{R_{b}}\right)}^{-\frac{1}{2}}\alpha_{nab}^{\frac{1}{2}} \end{array}\right.$$

where $k_{nab}$ is the normal elastic coefficient (or stiffness coefficient) of elements *a* and *b*; $E_{a}$ and $E_{b}$ are the elastic moduli of elements *a* and *b*, respectively. By the same token, the elastic moduli of elements *a* and *b* match those of sand particles *i* and *j*, i.e., $E_{a}=E_{i}$ and $E_{b}=E_{j}$.

When tangential micro-slip is neglected, according to Coulomb’s law of friction, the tangential contact force can be written as follows:

(8) $${\vec{F}}_{tc\_ab}=-\mu_{s}\left|{\vec{F}}_{nc\_ab}\right|\frac{{\vec{u}}_{tab}}{\left|{\vec{u}}_{tab}\right|}$$

where $\mu_{s}$ denotes the static friction coefficient between elements *a* and *b*, which is also the static friction coefficient between sand particles *i* and *j*; ${\vec{u}}_{tab}$ represents the relative tangential slip velocity at the contact interface between elements *a* and *b*.

During the 3DSP printing process, the direction of the tangential contact force does not necessarily align with that of the relative tangential slip velocity at the contact point. The tangential slip velocity vector may thus be expressed as follows:

(9) $${\vec{u}}_{tab}={\vec{u}}_{ij}-\left({\vec{u}}_{ij}\times \vec{n}\right)\vec{n}+R_{i}{\vec{\omega }}_{i}\times \vec{n}+R_{j}{\vec{\omega }}_{j}\times \vec{n}$$

where ${\vec{u}}_{ij}$ denotes the translational velocity of particle *i* relative to particle *j* with ${\vec{u}}_{ij}={\vec{u}}_{i}-{\vec{u}}_{j}$; $R_{i}$ and $R_{j}$ are the radii of sand particles *i* and *j*, respectively; ${\vec{\omega }}_{i}$ and ${\vec{\omega }}_{j}$ represent the angular velocities of sand particles *i* and *j*, respectively.

Drawing on the findings from Grimad [37], the tangential damping force ${\vec{F}}_{td\_ab}$ and normal damping force ${\vec{F}}_{nd\_ab}$ between elements *a* and *b* can be described as follows:

(10) $$\left\{\begin{array}{l} {\vec{F}}_{td\_ab}=\sqrt{\frac{10}{3}}\;\zeta {\left(k_{tab}m_{eq\_ab}\right)}^{\frac{1}{2}}{\vec{u}}_{tab}\\ {\vec{F}}_{nd\_ab}=\sqrt{5}\;\zeta {\left(k_{nab}m_{eq\_ab}\right)}^{\frac{1}{2}}{\vec{u}}_{nab} \end{array}\right.$$

where $m_{eq\_ab}$ denotes the equivalent mass of elements *a* and *b*, with $m_{eq\_ab}={\left(\frac{1}{m_{a}}+\frac{1}{m_{b}}\right)}^{-1}$; $m_{a}$ and $m_{b}$ are the masses of elements *a* and *b*, respectively; ${\vec{u}}_{tab}$ and ${\vec{u}}_{nab}$ represent the relative tangential slip velocity and relative normal velocity between elements *a* and *b*, respectively; ζ is the damping ratio, which is expressed as follows:

(11) $$\zeta =\frac{\ln e}{\sqrt{\ln^{2}\;e+\pi^{2}}}$$

where *e* is the coefficient of restitution of the sand particles.

The drag force ${\vec{F}}_{Di}$ exerted on sand particle *i* refers to the resistance parallel to the particle’s direction of motion when it moves in a fluid, and it is correlated with the fluid velocity. During the 3DSP sand-recoating process, air flow is nearly absent. Consequently, the drag force ${\vec{F}}_{Di}$ is negligible and can be neglected from calculations.

As indicated by Equations (6) and (7), both the tangential elastic coefficient $k_{tab}$ and normal elastic coefficient $k_{nab}$ are dependent on the normal overlap $\alpha_{nab}$ during sand-recoating. In theory, this would require real-time computation of these coefficients based on the contact process. Nevertheless, given the small magnitude of the normal overlap $\alpha_{nab}$—and for the sake of enhancing computational efficiency—it is assumed that the elastic and damping coefficients remain constant throughout the process, and the sand particle deformation is neglected.

The total tangential force ${\vec{F}}_{tij}$ and total normal force ${\vec{F}}_{nij}$ exerted on sand particle *i* by sand particle *j* are expressed, respectively, as follows:

(12) $$\left\{\begin{array}{l} {\vec{F}}_{tij}=\sum_{a=1}^{N_{i}}\left[\sum_{b=1}^{N_{j}}\left({\vec{F}}_{tc\_ab}+{\vec{F}}_{td\_ab}\right)\right]\\ {\vec{F}}_{nij}=\sum_{a=1}^{N_{i}}\left[\sum_{b=1}^{N_{j}}\left({\vec{F}}_{nc\_ab}+{\vec{F}}_{nd\_ab}\right)\right] \end{array}\right.$$

Since the normal force ${\vec{F}}_{nij}$ is directed toward the center of mass of sand particle *i*, it induces no torque on sand particle *i*, i.e., ${\vec{T}}_{nij}=0$. Accordingly, the total torque $T_{i}$ acting on sand particle *i* is given by the following equation:

(13) $$\left\{\begin{array}{l} {\vec{F}}_{tij}=\sum_{a=1}^{N_{i}}\left[\sum_{b=1}^{N_{j}}\left({\vec{F}}_{tc\_ab}+{\vec{F}}_{td\_ab}\right)\right]\\ {\vec{F}}_{nij}=\sum_{a=1}^{N_{i}}\left[\sum_{b=1}^{N_{j}}\left({\vec{F}}_{nc\_ab}+{\vec{F}}_{nd\_ab}\right)\right] \end{array}\right.$$

## 3. Experiment

### 3.1. Simulation Method and Parameter Configuration

**Figure 4:**
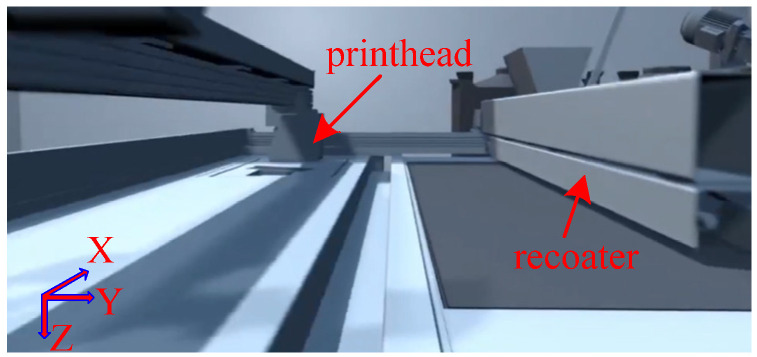
Schematic diagram of the build chamber of the EXONE S-MAX printer.

In this study, the simulation was conducted based on the EXONE S-MAX printer (North Huntingdon, PA, USA), which features a maximum forming dimension of 1.8 × 1 × 0.7 m^3^. As shown in Figure 4, the X-axis aligns with the printhead’s movement direction, the Y-axis corresponds to the recoater’s movement direction, and the Z-axis represents the sand mold’s movement direction. The curing agent was pre-mixed into the base sand which is composed of silica sand and ceramsite sand at a specified weight percentage.

- Resolution X: Defined as the distance the printhead travels between consecutive binder spraying events. A smaller value of Resolution X leads to increased binder deposition.
- Recoating speed: Refers to the linear velocity of the recoater along the Y-axis during operation.
- Layer thickness: Represents the thickness of the sand bed formed in a single pass of the recoater. During operation, sand is discharged from the gaps of the recoater via vibration. Upon completing one sand-recoating cycle, the recoater returns to its initial position to prepare for the subsequent cycle.

Three-dimensional models of the 3DSP printing system and sand particles were constructed using SolidWorks. For contact modeling, the Hertz–Mindlin (no slip) with RVD Rolling Friction model was employed to describe interactions between sand particles, and the Hertz–Mindlin (no slip) model was adopted for contact between sand particles and the printer’s mechanical components. The DEM was utilized to simulate the mechanical behaviors of irregular and spherical sand particles during the actual sand-recoating process. This approach enabled the analysis of how such behaviors affect the sand-recoating process, thereby facilitating the optimization of 3DSP process parameters.

(1) To ensure the reliability and high accuracy of the sand-recoating simulation, detailed and comprehensive modeling of the sand-recoating equipment is required. However, EDEM software cannot achieve the required modeling precision. Thus, SolidWorks was utilized to construct a 1:10 scale simulation model of the recoater and workbench involved in the sand-recoating process. Specifically, the workbench model has dimensions of 180 × 100 × 70 mm^3^.
(2) The selection of sand particle size is critical to achieving optimal sand-recoating performance. If the particle size is excessively large, sand particles exhibit high fluidity, but the density of the deposited sand layer decreases, which undermines printing accuracy. Conversely, while smaller particle sizes allow for thinner printed layers and higher precision, they intensify the van der Waals forces between particles. This causes agglomeration during sand-recoating, which impairs surface precision. To enhance sand-recoating accuracy, it is essential to blend sand particles of different sizes to fill gaps, ultimately forming a flatter and denser sand layer.

In this study, mixed sand composed of silica sand and ceramsite sand was employed, with the following specifications for each component:

- Ceramsite sand: Particles are relatively round and spherical. For the simulation, spherical particles following a normal distribution were adopted, with an average particle diameter of 0.15 mm.
- Silica sand: Particles are highly irregular with a large angularity coefficient. SolidWorks was used to model the irregular morphology of silica sand particles. Subsequently, EDEM’s multi-sphere filling method was employed to fill the 3D model of silica sand particles, enabling accurate simulation of their irregular shape. The average diameter of the irregular silica sand particles is 0.151 mm.

**Figure 5:**
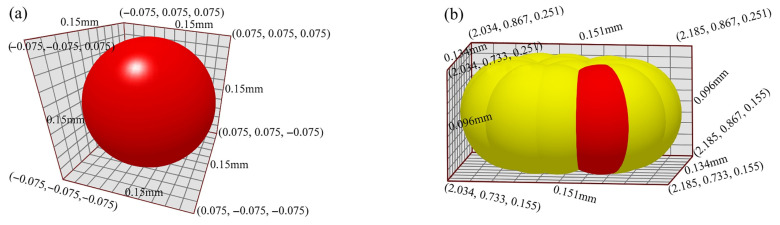
Established sand particle models: (**a**) spherical particles (ceramsite sand), (**b**) irregular particles (silica sand) based on the multi-sphere filling method.

The models of spherical and irregular sand particles are shown in Figure 5.

The recoater model, workbench model, sand particle model, and contact model were imported into EDEM. Following parameter calibration and processing, the integrated sand-recoating structure for the simulation was obtained. The physical properties of the sand and recoater materials in the study are presented in Table 1. The interaction parameters among different materials are presented in Table 2 [24].

Previous research [38] has indicated that parameters including resolution X, layer thickness, curing agent content, sand-discharge port width, and sand-scraper angle exert a significant influence on the mechanical properties of 3DSP samples. Consequently, when investigating the effects of the silica sand/ceramsite sand ratio and the recoating speed on the sand-recoating process, the above parameters were maintained constant. The process parameters for the simulation experiments are presented in Table 3. Each experiment was simulated five times and the Grubb’s test was adopted (confidence level: 95%, corresponding critical value *G*_0.05, 5_ = 1.672) to eliminate outliers. The average value of the remaining valid data, along with the standard deviation, was used as the final result.

### 3.2. Experimental Materials and Methods

Mixed sand composed of silica sand and ceramsite sand was used in this experiment, with a mesh size of 70–140 (corresponding to a particle size range of 0.106–0.212 mm). Furfuryl resin (with viscosity of 8–14 mPa·s and density of 1.1–1.2 g·cm^−3^) was used as the binder, while toluenesulfonic acid (with viscosity of 10–20 mPa·s and density of 1.2–1.3 g·cm^−3^) was used as the curing agent.

**Figure 6:**
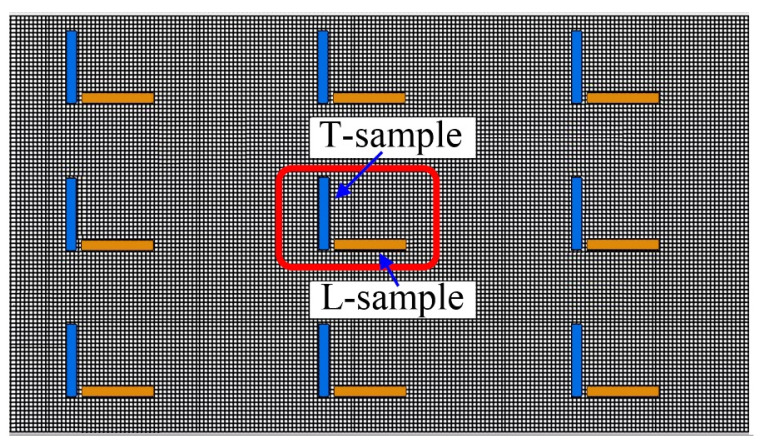
Schematic diagram of the layout of bending strength specimens (dimensions: 22.4 × 22.4 × 172 mm^3^) on the workbench of EXONE S-MAX printer.

Specimens for bending strength testing were printed using the EXONE S-MAX printer, with dimensions of 22.4 × 22.4 × 172 mm^3^ which complies with the requirements for GB/T 2684–2025 [39]. The layout of bending strength specimens on the EXONE S-MAX workbench is illustrated in Figure 6. The specimens were categorized into two types: longitudinal specimens (L-samples) and transverse specimens (T-samples). Printing was conducted in accordance with the process parameters detailed in Table 3, aiming to verify the simulation results of silica sand/ceramsite sand ratio and recoating speed, respectively. After printing, the specimens were allowed to stand at room temperature (20–25 °C) for 24 h prior to the execution of mechanical property tests. For each experimental group, 9 L-samples and 9 T-samples were printed.

The printed specimens were subjected to bending properties testing via a three-point bending test on an XQY-II intelligent sand strength tester (Wuxi Sanfeng Instruments & Equipment Co., Ltd., Wuxi, China) with an accuracy of 1.0%, in compliance with GB/T 2684–2025. For 9 experimental measurements, the Grubb’s test was adopted (confidence level: 95%, corresponding critical value *G*_0.05, 9_ = 2.110) to eliminate outliers. The average value of the remaining valid data, along with the standard deviation, was used as the final result.

## 4. Results and Discussions

### 4.1. Effect of Sand Ratio on Sand-Recoating Performances and Mechanical Properties

**Figure 7:**
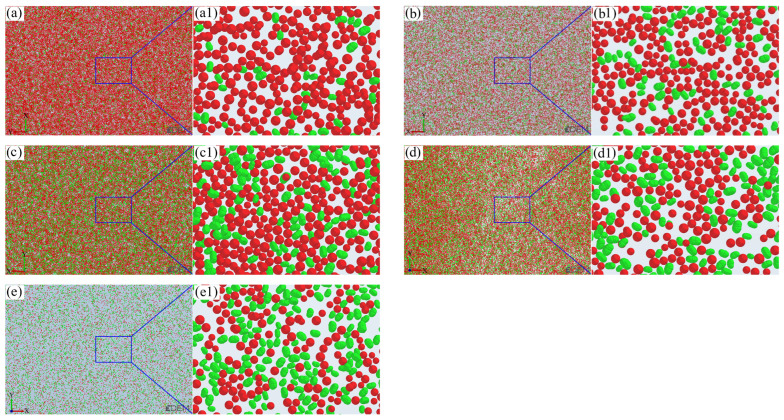
Top views of sand-recoating performances under different ratios of silica sand to ceramsite sand (red: ceramsite sand, green: silica sand): (**a**) 1:9, (**b**) 2:8, (**c**) 3:7, (**d**) 4:6, (**e**) 5:5, (**a1**–**e1**) corresponding to the magnified views of the layouts in (**a**–**e**), respectively.

The sand-recoating performances achieved with different mixing ratios of silica sand and ceramsite sand are presented in Figure 7. Here, silica sand is depicted in green, ceramsite sand in red, and the workbench in gray.

As observed in Figure 7, silica sand and ceramsite sand exhibit different particle sizes, which corresponds to the sand particle diameter range of 0.106–0.212 mm specified in the simulation parameter settings. With an increase in the ratio, the packing density of sand particles first increases and then decreases. The degree of compactness between sand particles can be characterized by porosity, which is calculated based on the volume ratio of the gray area in the figure. The calculation formula is as follows:

(14) $$P=1-\frac{V_{a}+V_{\mathrm{as}}}{l\cdot h\cdot d}$$

where *P* denotes the porosity, *V*_a_ and *V*_as_ represent the volume of virtual sand particles and curing agent, respectively, and *l*, *h* and *d* are the length, height and thickness of the sand bed.

**Figure 8:**
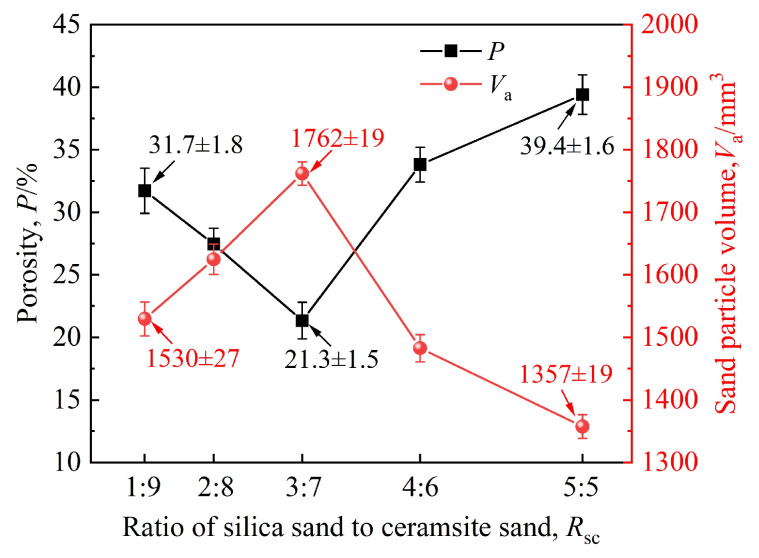
Variations in porosity and sand particle volume with the ratio of silica sand to ceramsite sand.

During the simulation, bonding bonds were used to replace the curing agent, meaning *V*_as_ can be neglected. The parameters were set as follows: *l* = 100 mm, *h*= 80 mm, and *d*= 0.28 mm. Using the sand particle volume data derived from the simulation results, the porosity under different silica sand/ceramsite sand ratios can be calculated via Equation (14), and the variations in porosity and sand particle volume are shown in Figure 8.

As depicted in Figure 8, with the progressive increase in the ratio of silica sand to ceramsite sand, the porosity exhibits a trend of first decreasing and then increasing, whereas the total sand particle volume exhibits the opposite pattern of initial increase and then decrease. When the ratio rises from 1:9 to 3:7, the total sand particle volume increases from 1530 mm^3^ to 1762 mm^3^, with a corresponding reduction in porosity from 31.7% to 21.3%. As the ratio further increases to 5:5, the total sand particle volume drops from 1762 mm^3^ to 1357 mm^3^, accompanied by a rise in porosity from 21.3% to 39.4%.

**Figure 9:**
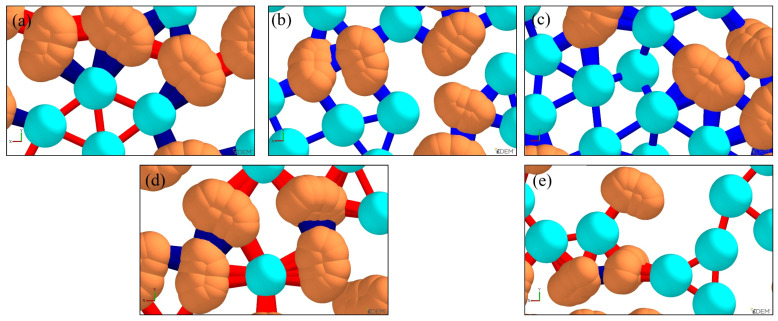
Simulation results of bonding bridges (red and blue) under different ratios of silica sand (light green) to ceramsite sand (yellow): (**a**) 1:9, (**b**) 2:8, (**c**) 3:7, (**d**) 4:6, (**e**) 5:5.

Owing to the high angularity coefficient and poor fluidity of silica sand, bridging is prone to occur during the sand-recoating process. Furthermore, as silica sand content increases, the bridging effect becomes more severe, resulting in higher porosity. However, when the silica sand content is low (the ratio < 3:7), the ceramsite sand content is relatively high. The smaller-sized spherical ceramsite sand particles can infiltrate the gaps formed by inter-particle bridging, occupying portions of existing pores and thereby reducing porosity. When the ratio reaches 3:7, the porosity attains a minimum of 21.3%, with the corresponding total sand particle volume *V_a_* peaking at 1762 mm^3^. Beyond this ratio, a further increase in the silica sand proportion leads to insufficient ceramsite sand to fill additional pores. Additionally, the excessive ceramsite sand accumulates in a spherical particle packing manner, creating more voids and consequently increasing the porosity. This porosity variation pattern with the ratio is also evident in Figure 9. Thus, when the ratio of silica sand to ceramsite sand is 3:7, the porosity is minimized, the sand bed density is maximized, and the sand-recoating performance is optimal.

After rendering sand particles and bonding bridges using EDEM’s post-processing functionality, the comparative analyses of bonding bridges under different ratios were obtained, as shown in Figure 9. Here, light green denotes ceramsite sand, yellow denotes silica sand, and red and blue denote bonding bridges. Specifically, blue bonding bridges indicate a greater shear force capacity between sand particles, while red bonding bridges indicate a lower shear force capacity.

As observed in Figure 9, the ratio exerts a significant influence on the bonding bridges between sand particles. As the sand porosity decreases, the contact area between sand particles increases. Consequently, both the quantity and cross-sectional area of inter-particle bonding bridges rise, which in turn enhances the mechanical properties of the molding sand. At a ratio of 3:7, the porosity reaches its minimum, and the corresponding quantity and cross-sectional area of bonding bridges are maximized, as illustrated in Figure 9c. Beyond this ratio, further increases in the ratio lead to a gradual reduction in the quantity and cross-sectional area of bonding bridges. It can thus be concluded that when the ratio of silica sand to ceramsite sand is 3:7, the bonding bridges between sand particles exhibit the highest quantity and largest cross-sectional area, and the molding sand achieves optimal mechanical properties.

**Figure 10:**
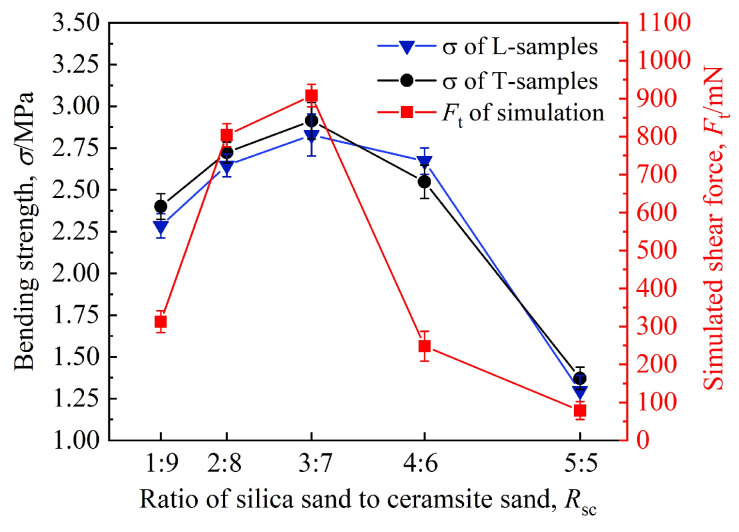
Variations in simulated shear force and bending strength with the ratio.

Figure 10 presents the simulation results of the critical shear force sustainable by sand particles, together with the experimentally measured bending strength, both plotted as functions of the ratio. As observed, with an increase in the ratio of silica sand to ceramsite sand, the critical shear force first rises rapidly from 313 mN at a ratio of 1:9 to a maximum of 908 mN at a ratio of 3:7, then declines sharply to 248 mN, and ultimately drops to a minimum of 79 mN when the ratio reaches 5:5. This variation trend of critical shear force aligns with the changes in bonding bridges observed in Figure 10.

Figure 10 further reveals that the bending strength of L-samples and T-samples follows an almost identical variation pattern. This is primarily because both specimens were fabricated under identical experimental conditions. The slight differences in bending strength between the two specimens are mainly ascribed to their distinct molding orientations on the workbench. The variation trend of the experimentally measured bending strength of the specimens shows good agreement with that of the simulated shear force—both first increase and then decrease with the increasing ratio. At a ratio of 3:7, the bending strength of L-samples and T-samples reaches their maximum values of 2.83 MPa and 2.91 MPa, respectively, with an average of 2.87 MPa.

**Figure 11:**
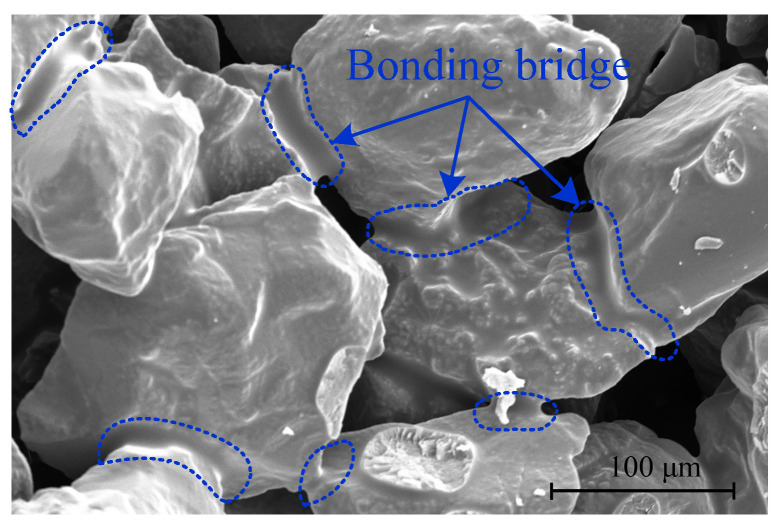
SEM images of bonding bridges [38].

The strength of molding sand is conferred by the bonding bridges formed between adjacent sand particles. These bridges arise from the polymerization of resin coating the sand particle surfaces, with the curing agent acting as a catalyst. The microscopic morphology of these bonding bridges is illustrated in Figure 11 [38]. Notably, the strength of molding sand is closely associated with both the quantity of bonding bridges and the strength of individual bonding bridges.

As observed in Figure 11, the bonding bridges between sand particles are irregular in shape, and their cross-sectional area and distribution density are directly determined by the sand particle packing state. When the ratio is 3:7, the optimized particle gradation results in the smallest porosity of 21.3%, leading to the largest contact area between adjacent particles. This promotes the uniform spread and penetration of the furfuryl resin under capillary force, forming bonding bridges with a dense structure and large cross-sectional area in Figure 9c.

**Figure 12:**
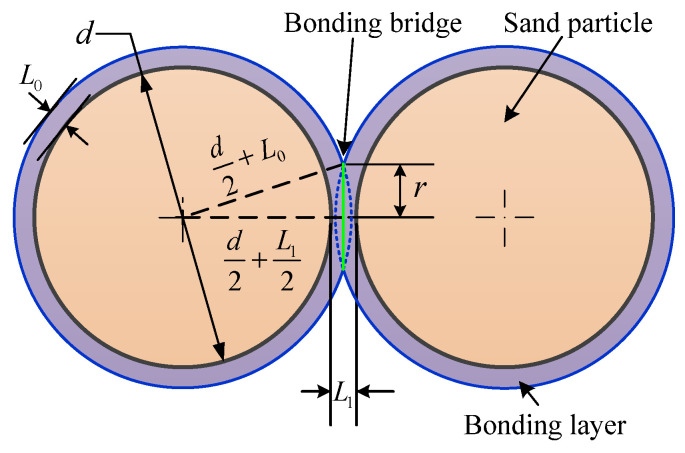
Schematic diagram of bonding bridges between adjacent sand particles.

For simplicity, the sand particles are assumed to be spherical and arranged in a simple regular configuration. Accordingly, the bonding bridge model between two sand particles can be simplified as illustrated in Figure 12 [40].

Assuming the sand particles have a diameter of *d*, the thickness of bonding layer adhering to the sand particle surfaces is *t*, the strength of an individual bonding bridge after binder curing is $\sigma_{0}$, the minimum cross-sectional area of a bonding bridge is $S_{n}$, and the distance between bonding bridges is *L*_1_, it is easy to derive $0<L_{1}<2L_{0}$. Accordingly, the molding sand strength σ can be expressed as follows:

(15) $$\sigma =\frac{N_{1}\sigma_{0}S_{n}}{S}=\frac{N_{1}\sigma_{0}\pi \left[{\left(\frac{d}{2}+L_{0}\right)}^{2}-{\left(\frac{d}{2}+\frac{L_{1}}{2}\right)}^{2}\right]}{S}$$

where *S* denotes the cross-sectional area of the specimen’s fracture surface, *N*_1_ represents the number of bonding bridges on the cross-section of the sand specimen, which can be characterized using the packing density *ρ* of sand particles and the density $\rho_{0}$ of a single sand particle, i.e., the following:

(16) $$N_{1}=\frac{6S}{\pi d^{2}}\frac{\rho }{\rho_{0}}$$

The thickness $L_{0}$ of the binding layer on the sand particle surfaces is related to the resin content *w*, and their relationship can be expressed as follows [41]:

(17) $$L_{0}=\sqrt[{3}]{\frac{3w}{4\pi }+{\left(\frac{d}{2}\right)}^{2}}-\frac{d}{2}$$

Substituting Equations (16) and (17) into Equation (15), and setting *L*_1_ = *L*_0_, the following equation is derived:

(18) $$\sigma =\sigma_{0}\frac{\rho }{\rho_{0}}\left[{\left(\frac{3}{2\sqrt{2}}\sqrt[{3}]{\frac{6w}{\pi d^{3}}+1}-\frac{1}{2\sqrt{2}}\right)}^{2}-\frac{1}{2}\right]$$

Equation (18) demonstrates that when the resin content *w*, sand particle diameter *d*, and density of individual sand particles *ρ*_0_ are held constant, the sand mold strength depends only on the strength of individual bonding bridge $\sigma_{0}$ and the sand particle packing density *ρ*.

The packing density of sand particles is inversely proportional to porosity. As derived from the results in Figure 8, the sand particle packing density first increases and then decreases with variations in the ratio, reaching its maximum value when the ratio is 3:7. With an increase in the ratio, the color of the bonding bridges in Figure 9 transitions from red to blue and then back to red. This color shift indicates that the strength of the bonding bridges follows a corresponding trend of first increasing and then decreasing, with the maximum strength observed at a ratio of 3:7. Consequently, under the combined influence of sand particle packing density and bonding bridge strength, the bending strength of the specimens, as measured experimentally, exhibits a trend of first increasing and then decreasing, with its maximum value attained at a ratio of 3:7.

Furthermore, Equation (15) also indicates that the macroscopic strength of the specimens is positively correlated with two key parameters: the number *N*_1_ of bonding bridges and the strength *σ*_0_ of individual bonding bridge. When the ratio is below 3:7, the high proportion of ceramsite sand results in small inter-particle gaps, which limits furfuryl resin penetration and thus forms thin, sparse bonding bridges with low strength *σ*_0_. When the ratio is more than 3:7, excessive silica sand causes severe bridging, leading to an increased porosity, decreased packing density, less bonding bridges *N*_1_. Insufficient bonding between particles also lowers the strength *σ*_0_ of individual bonding bridges. Thus, the “first increase then decrease” trend of macroscopic strength is essentially a reflection of the synergistic effect of the number and strength of bonding bridges at the microscale. In the DEM simulation, the shear force of bonding bridges is quantified by calculating the contact force between particle elements. When the ratio is 3:7, the simulated shear force reaches 908 mN, corresponding to the maximum values of *N*_1_ and *σ*_0_. The experimentally measured bending strength is 2.87 MPa, which is consistent with the simulation results.

### 4.2. Effect of Recoating Speed on Sand-Recoating Performances and Mechanical Properties

**Figure 13:**
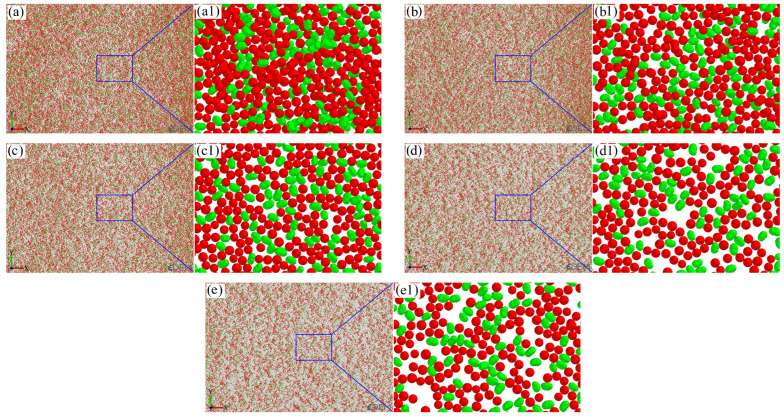
Top views of sand-recoating performances under different recoating speeds (red: ceramsite sand, green: silica sand): (**a**) 120 mm·s^−1^, (**b**) 140 mm·s^−1^, (**c**) 160 mm·s^−1^, (**d**) 180 mm·s^−1^, (**e**) 200 mm·s^−1^, (**a1**–**e1**) corresponding to the magnified views of the layouts in (**a**–**e**), respectively.

The sand-recoating performances achieved with different recoating speeds are presented in Figure 13. Here, silica sand is depicted in green, ceramsite sand in red, and the workbench in gray.

**Figure 14:**
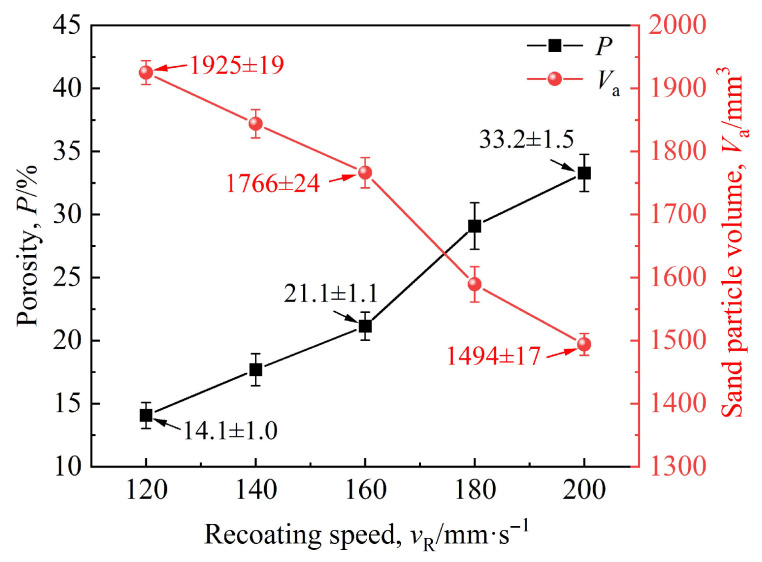
Variations in porosity and sand particle volume with the recoating speed.

As observed in Figure 13, the packing density of sand particles exhibits a consistent decreasing trend with the increasing recoating speed. It reaches a maximum at a recoating speed of 120 mm·s^−1^ and a minimum at 200 mm·s^−1^. A calculation domain with parameters *l* = 100 mm, *h* = 80 mm, and *d* = 0.28 mm was selected. The sand particle volume data were exported, and the porosity values were calculated using Equation (14). The variations in relationships between sand particle volume, porosity, and recoating speed are presented in Figure 14.

Figure 14 shows the total sand particle volume decreases consistently with a continuous increase in recoating speed, declining from 1925 mm^3^ at 120 mm·s^−1^ to 1494 mm^3^ at 200 mm·s^−1^. In contrast, the porosity increases continuously with the increasing recoating speed, rising from 14.1% at 120 mm·s^−1^ to 33.2% at 200 mm·s^−1^. At a recoating speed of 160 mm·s^−1^, the total sand particle volume is 1766 mm^3^ while the porosity is 21.1%.

The above trends can be explained as follows. At lower recoating speeds, the recoater moves slowly. With the vibration frequency and sand-discharge port width remaining constant, a longer recoating duration allows more sand particles to be vibrated onto the workbench. During the falling process of the sand particles, the scraper continuously levels the sand, resulting in a higher sand density per unit area on the workbench and a denser sand layer. As the recoating speed increases, the recoater moves faster, reducing the amount of sand vibrated onto the workbench and thereby increasing porosity. When the recoating speed reaches a certain threshold, the sand vibrated onto the workbench becomes relatively more dispersed, leading to a further increase in porosity.

**Figure 15:**
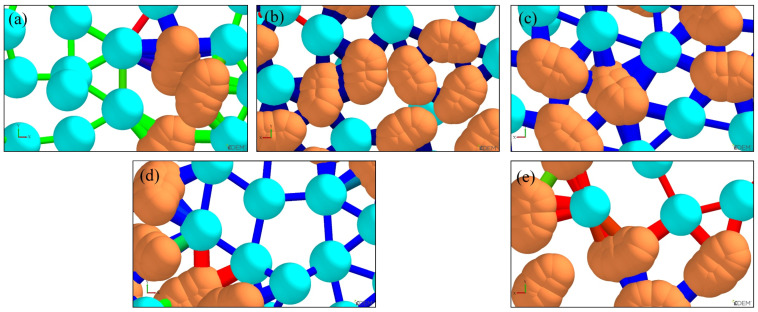
Comparison of simulated bonding bridges under different recoating speeds (blue bonding bridges have the maximum shear force, green bonding bridges have the second-highest shear force, and red bonding bridges have the minimum shear force): (**a**) 120 mm·s^−1^, (**b**) 140 mm·s^−1^, (**c**) 160 mm·s^−1^, (**d**) 180 mm·s^−1^, (**e**) 200 mm·s^−1^.

The bonding bridges between sand particles were rendered using the post-processing function of EDEM, and a comparison of these bonding bridges under different recoating speeds is presented in Figure 15. Here, blue bonding bridges indicate the maximum shear force sustainable between sand particles, green bonding bridges represent the second-highest sustainable shear force, and red bonding bridges denote the minimum sustainable shear force.

Figure 15 demonstrates that sand-recoating speed has a significant impact on the strength and quantity of bonding bridges between sand particles. As the recoating speed increases, the color of the bonding bridges transitions from green to blue, and subsequently to red.

At a recoating speed of 120 mm·s^−1^, the major bonding bridges are green and relatively slender, with a small number of blue and red bonding bridges also present. When the speed reaches 140 mm·s^−1^, the green bonding bridges disappear, the number of blue bonding bridges increases, and only a few red bonding bridges can be observed, accompanied by a substantial increase in shear force. At a recoating speed of 160 mm·s^−1^, all bonding bridges turn blue and are relatively abundant and thick, thus the shear force between sand particles reaches its maximum value, corresponding to the optimal mechanical properties of the sand. As the recoating speed continues to rise, blue bonding bridges become thin, while red and green bonding bridges reemerge, resulting in a reduction in shear force. At a recoating speed of 200 mm·s^−1^, the total number of bonding bridges becomes extremely low, and most of them are red, which can only sustain low shear forces. Therefore, from the perspective of bonding bridge performance, a recoating speed of 160 mm·s^−1^ is recommended.

**Figure 16:**
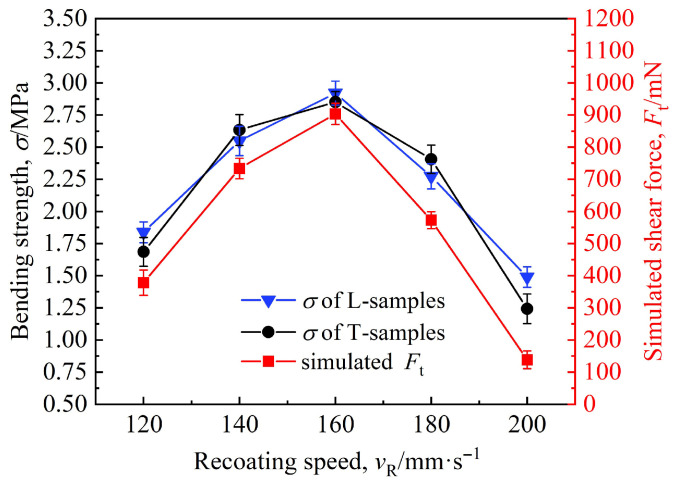
Variations in simulated shear force and bending strength with the recoating speed.

Figure 16 presents the simulated results of the shear force sustainable between sand particles and the experimentally measured bending strength, both plotted as functions of the recoating speed.

As observed in Figure 16, both the shear force and bending strength exhibit a trend of first increasing and then decreasing with the increase in recoating speed. When the recoating speed rises from 120 mm·s^−1^ to 160 mm·s^−1^, the shear force and bending strength increase in tandem with the increasing speed. Specifically, the shear force of the bonding bridges increases from 378 mN to a maximum of 903 mN, while the bending strengths of the L-samples and T-samples increase from 1.84 MPa and 1.69 MPa to 2.92 MPa and 2.85 MPa, respectively (with an average of 2.89 MPa), representing the peak values under the experimental conditions. Thereafter, as the recoating speed continues to increase, the shear force of the bonding bridges and the bending strength of the samples decrease rapidly. At a recoating speed of 200 mm·s^−1^, both the shear force and average bending strength reach their minimum values of 138 mN and 1.37 MPa (with the L-samples and T-samples measuring 1.49 MPa and 1.24 MPa, respectively). On the basis of these results, the optimal recoating speed is determined to be 160 mm·s^−1^, which is basically consistent with Sivarupan’s research results on the optimal recoating speed for single-type sand [19].

The variation trend of bending strength—first increasing and then decreasing—is highly consistent with Coniglio’s findings regarding the bending strength of single-type sand as a function of recoating speed [20]. However, the bending strength (1.24–2.92 MPa) in this study is significantly higher than that reported in Coniglio’s research (1.12–2.76 MPa). This is primarily attributed to the use of mixed sand, confirming that mixed sand can yield superior mechanical properties.

The variation can be explained as follows. The accumulated sand particles can be regarded as a porous structure. The binder jetted onto the sand surface will spread and wet the surface. Subsequently, under capillary force, the spread binder will penetrate into the deeper layers of sand particles. The penetration depth of the binder is given by the following equation [42]:

(19) $$D_{p}=\frac{r_{s}\cdot \gamma_{b}\cdot \cos\theta }{4\mu_{b}\cdot \nu_{p}}$$

where $D_{p}$ denotes the penetration depth of the binder, $r_{s}$ represents the radius of pores between sand particles, $\gamma_{b}$ is the surface tension of the binder, *θ* stands for the contact angle between the binder and the pores of sand particles, $\mu_{b}$ denotes the viscosity of the binder, and $\nu_{p}$ is the speed at which the binder reaches the lower sand surface.

At low recoating speeds, sand particles within the sand-recoating plane are densely packed. While Equation (18) would imply relatively high bending strength, the actual strength of the sand is derived from the polymerization of the binder. When sand particles are densely packed, the inter-particle porosity is low and the pore radius $r_{s}$ is small. As indicated by Equation (19), this leads to a shallow penetration depth of the binder, insufficient to effectively infiltrate the gaps between adjacent sand particles. Such inadequate binder diffusion hinders polymerization of the binder, ultimately forming fewer and slimmer bonding bridges, as shown in Figure 15a. This in turn leads to low shear force of the bonding bridges and reduced bending strength of the specimens.

As the recoating speed increases, the packing density of sand particles in the sand-recoating plane decreases, accompanied by an increase in porosity and a larger inter-particle pore radius $r_{s}$. Driven by capillary force, the binder’s penetration capability between sand particles is enhanced, resulting in thicker and more numerous bonding bridges (all blue in Figure 15c), which causes an increase in shear force and tensile strength. At a recoating speed of 160 mm·s^−1^, the maximum shear force of 903 mN combined with an optimal number of bonding bridges *N*_1_ (or optimal packing density *ρ*) jointly results in a peak strength of 2.89 MPa.

Beyond this optimal speed, a further increase in recoating speed causes porosity to rise and packing density to decline, reducing the bending strength of the specimens in accordance with Equation (18).

When the inter-particle pore radius $r_{s}$ exceeds a certain threshold, the excessive gaps between sand particles significantly impair the binder’s ability to penetrate via capillary force. At this stage, the binder penetration depth can no longer be characterized by Equation (19). Deprived of the driving force from capillary action, the binder’s penetration depth drops sharply, forming sparse and fragile bonding bridges (mostly red in Figure 15e). Both the quantity and cross-sectional area of inter-particle bonding bridges shrink drastically, leading to a subsequent reduction in the shear force of the bonding bridges and the bending strength of the sand specimens.

### 4.3. Model Limitations and Impacts of Simplifying Assumptions

To balance computational efficiency and engineering practicality, three simplifying assumptions were integrated into the development of the DEM-based 3DSP recoating process model. Below is a concise analysis of their impacts on result accuracy and associated model limitations, integrated with physical mechanisms and quantitative evidence:

(1) **Impact of constant elastic and damping coefficients**: In the model, the normal and tangential elastic coefficients, as well as damping coefficients for sand particles were assumed to be constant. Their dynamic variations with particle contact states (e.g., normal overlap, contact angle) were not also taken account. From a physical standpoint, mechanical parameters at the sand particle contact interface may exhibit slight fluctuations induced by local pressure differences during actual recoating. However, according to the relevant literature [43], the normal overlap of sand particles is consistently less than 5% of the particle diameter, and the impact of these fluctuations on particle packing structure and contact force distribution is negligible—far below the relative standard deviation of repeated simulation results. Thus, while this assumption introduces slight systematic deviations in contact force calculations, it does not alter the core regulatory pattern that “the mixing ratio/recoating speed and performance exhibit a trend of first increasing and then decreasing,” nor does it affect the identification of the optimal parameter combination (mixing ratio 3:7 and recoating speed 160 mm·s^−1^). Consequently, it poses no substantial compromise to the validity of the results.
(2) **Impact of neglecting particle deformation**: Silica sand and ceramsite sand particles are treated as rigid bodies in the model, with microscopic deformation being neglected. In terms of material properties, both silica sand (Mohs hardness 7) and ceramsite sand (Mohs hardness 6.5) have a compressive strength exceeding 100 MPa, whereas the inter-particle contact pressure during recoating, derived from simulations, is only 0.1–0.5 MPa, is far lower than the yield strength of the sand particles. This leads to negligible actual particle deformation, which is negligible in engineering calculations. Experimental validation demonstrates good consistency between simulated shear force and measured bending strength, further confirming that this assumption does not significantly compromise the model’s accuracy in predicting mechanical properties. Slight deviations may only arise under extreme high-pressure conditions.
(3) **Impact of neglecting drag force**: The model does not account for the viscous drag force exerted by air on sand particles. The 3DSP recoating process in this study involves low-speed mechanical spreading, with a maximum speed of 200 mm·s^−1^. Furthermore, the density of sand particles (2650–3030 kg/m^3^) is considerably higher than that of air (1.29 kg/m^3^). Based on Stokes’ law, the drag force acting on sand particles is only 0.01–0.03% of their self-weight, which is significantly smaller than the inter-particle contact force. Thus, neglecting drag force does not alter the motion trajectories or packing morphology of sand particles, nor does it exert a meaningful impact on the calculation deviations of key indicators (e.g., porosity, shear force). This fully satisfies the precision requirements for engineering simulations.

Based on the above analysis, under the existing simplifying assumptions, the core applicable scenarios of the established model are confined to the common operating conditions for 3DSP, specifically defined as follows: recoating speed ≤ 250 mm·s^−1^ (falling within the low-speed recoating range), sand particle size of 0.1–0.3 mm (covering the conventional industrial sand particle size interval), and a room-temperature, atmospheric-pressure environment.

Within the applicable boundaries, the core conclusions of the model demonstrate exceptional robustness: the mechanical properties exhibit a “first increase and then decrease” regulatory trend with variations in the mixing ratio or recoating speed, and the effectiveness of the optimal process parameter combination (a ratio of 3:7 coupled with a recoating speed of 160 mm·s^−1^) remains unaffected by the simplifying assumptions. The consistency between the simulation results and experimental data further validates the reliability of the model under these operating conditions.

If the operating conditions deviate beyond the above boundaries (e.g., sand particle size < 0.1 mm, recoating speed > 250 mm·s^−1^), the simplifying assumptions will lead to a significant decline in simulation accuracy. For fine-grained sand particles (<0.1 mm), the proportion of van der Waals forces between particles surges dramatically. Since the existing model does not account for this intermolecular force, deviations in the prediction of particle packing density and bonding bridge strength will be substantially amplified. During high-speed recoating (>250 mm·s^−1^), the interference of the air flow field and particle turbulence effects become increasingly prominent, rendering the original model’s assumptions of “neglecting drag force” and “uniform speed spreading” no longer valid. For such extreme operating conditions, the model optimization is imperative through the supplementation of correction terms: a van der Waals force correction equation should be incorporated for the fine particle size scenario, while CFD technology needs to be coupled to simulate gas–solid two-phase interactions for high-speed recoating.

## 5. Conclusions

(1) Based on the discrete element method, a contact constitutive model, an inter-particle contact force model, and a sand particle motion model for the sand-recoating process in 3DSP printing were established. The displacement of sand particles was calculated, and combined with the sand particle model, recoater model, and workbench model constructed using SolidWorks and EDEM; the simulation of the 3DSP sand-recoating process using sand particle ensemble was realized.
(2) The ratio of silica sand to ceramsite sand significantly regulates the particle packing structure and inter-particle bonding in 3DSP. With the increasing ratio, porosity first decreases and then increases, while mechanical properties (governed by the quantity and strength of bonding bridges) follow an opposite trend. The optimal ratio (3:7) achieves the densest sand bed and strongest bonding, offering direct guidance for industrial sand formulation to balance packing density and strength.
(3) Recoating speed exerts a substantial influence on porosity and inter-particle shear force in 3DSP process. The porosity increases consistently with the recoating speed, whereas the inter-particle shear force and mechanical properties first rise and then fall. The optimal speed (160 mm·s^−1^) strikes a balance between packing density and binder diffusion, ensuring superior mechanical properties.
(4) Experimental validation confirms that the DEM simulation results are well consistent with bending strength measurements, verifying the model’s reliability. It can be directly applied in parameter optimization of industrial 3DSP production to reduce trial-and-error costs and shorten development cycles. The optimized parameter combination (3:7 ratio + 160 mm·s^−1^ speed) can obtain maximum bending strength.

### Limitations and Future Work

This study developed a DEM-based model for the sand-recoating process in 3DSP, revealing the effects of the silica sand-to-ceramsite sand ratio and recoating speed on sand-recoating performance and mechanical properties. It thereby provided a fundamental support for the optimization of 3DSP process parameters. However, the study did not couple DEM with CFD, overlooking the coupling effects between fluids (binder and air) and particles, which makes it difficult to accurately reflect the binder penetration process. Additionally, the particle deformation and the dynamic polymerization of the curing agent was simplified to be constant mechanical parameters, leading to certain limitations in simulating complex conditions. Therefore, in future research, DEM can be integrated with CFD to improve the multi-field interaction model; the scope of parameters can be expanded to explore the interactive effects of multiple factors.

## Figures and Tables

**Table 1:** Physical properties of sand and recoater materials for simulation.

Materials	Poisson Ratio, *ν*	Shear Modulus, *G*/GPa	Elastic Modulus, *E*/GPa	Density, *ρ*/kg·m^−3^
ceramsite sand	0.25	60.2	150	3030
silica sand	0.16	24.5	57	2650
steel	0.30	79.4	206	7850

**Table 2:** Interaction parameters between materials during simulation.

Materials	Restitution Coefficient, *e*	Sliding Friction Coefficient	Static Friction Coefficient
ceramsite sand vs. ceramsite sand	0.3	0.05	0.6
ceramsite sand vs. steel	0.5	0.05	0.4
silica sand vs. silica sand	0.5	0.05	0.4
silica sand vs. steel	0.4	0.05	0.3
ceramsite sand vs. silica sand	0.4	0.05	0.5

**Table 3:** Process parameters of 3DSP experiments.

Parameters	Values	
Resolution X (mm)	0.09	0.09
Layer thickness (mm)	0.28	0.28
curing agent content (%)	0.14	0.14
sand-discharge port width (mm)	1.2	1.2
sand-scraper angle (°)	4	4
Vibration Frequency (Hz)	75	75
Recoating speed (mm·s^−1^)	160	120, 140, 160, 180, 200,
Ratio of silica sand to ceramsite sand	1:9, 2:8, 3:7, 4:6, 5:5	3:7

## Data Availability

The original contributions presented in this study are included in the article. Further inquiries can be directed to the corresponding authors.
